# Modelling the transport and deposition of sediment-microplastics fluxes in a braided river, using Delft3D

**DOI:** 10.1098/rsta.2024.0442

**Published:** 2025-10-23

**Authors:** Lucrecia Alvarez Barrantes, Anne Baar, Roberto Fernández, Christopher Hackney, Daniel Parsons, Robert M. Dorrell

**Affiliations:** ^1^ Geography Deparment, Loughborough University Faculty of Social Sciences and Humanities, Loughborough, UK; ^2^ Department of Water Management, Delft University of Technology Department of Geoscience & Engineering, Delft, Zuid-Holland, The Netherlands; ^3^ Department of Civil and Environmental Engineering, Penn State University, University Park Campus, State College, PA 16801, USA; ^4^ School of Geography, Politics and Sociology, Newcastle University - Newcastle Campus, Newcastle upon Tyne, UK; ^5^ Pro-Vice-Chancellor for Research and Innovation, Loughborough University, Loughborough, UK; ^6^ School of Architecture, Building and Civil Engineering, Loughborough University, Loughborough, UK

**Keywords:** rivers, microplastics, transport, deposition, hydromorphodynamic numerical model, erosion

## Abstract

Rivers polluted by plastics have become sites where mixtures of microplastics and sediment particles are transported by the river current and deposited in the riverbed. A hydromorphodynamic numerical model was developed using Delft3D (software specialized in simulating natural water systems), to simulate the sedimentation, erosion, resuspension and transport of microplastics together with sediment particles, introducing an innovative model with an active riverbed. The model was used to understand the distribution patterns, morphological changes and load balances of plastic debris in a river. The study case is an artificial braided river with a non-buoyant suspended microplastic load. The results simulate a sediment bed that acts as a source of microplastic storage near the point of release. The high deposition of microplastics increases the capacity of the river flow to erode the banks and channels, resulting in deeper channels and larger river bars. The highest amounts of microplastics were deposited in the inner channel banks, and the highly suspended microplastic load is transported in the main channel thalweg. The model can be used as a more accurate method to predict the dynamics of microplastic fluxes in rivers, providing better tools to understand how much plastic enters the ocean from the river environment.

This article is part of the Theo Murphy meeting issue ‘Sedimentology of plastics: state of the art and future directions’.

## 1. Introduction

Microplastic pollution (plastic particles smaller than 5000 µm) is a problem facing rivers, oceans, coastlines and deep-sea environments [1–3]. It is estimated that approximately 1.15–12.7 million tons of plastic waste enter the oceans annually from river systems [4,5]. The river plastic load is estimated to contribute 80% of the plastic in the oceans, making rivers the main source of plastic pollution [4–6]. Microplastics can contain chemicals associated with human diseases and can cause harmful effects to organisms [7–9]. Understanding the transport and depositional behaviour of microplastic particles is essential to better understand the amount of plastic stored and transported in the river system and to mitigate its effects.

Studies of microplastic pollution in rivers have determined that the plastics are deposited on and in the riverbed and transported by the flow, following the complex river process that depend on physical properties of the particles, hydraulics, hydrology, meteorological conditions, morphology and vegetation [10–20]. In the riverbed, microplastics form mixed layers with the sediments that are subject to remobilization, deposition and immobilization by long-term burial [21,22].

The study of the dynamics of microplastic transport and deposition have previously been incorporated into river flow models to understand the spatial and temporal distributions of this contaminant. These models combine the influences of tides, wind currents, bathymetry, sources and aggregation processes, using theories of river flow hydrodynamics, statistical and mass balance [23,24]. However, few studies integrate the effect of sediment and microplastic storage in the riverbed [21,22]. These models typically consider a static riverbed without integrating sediment–microplastic interactions. This lack of process representation means such models fail to account for the effect of plastics in increasing critical shear stresses that may be required for erosion [25,26]. Furthermore, it has been shown that plastics change the mean particle diameter of materials in the bed and affect the resulting bedforms [19], influencing the availability of plastic particles for transport [22,27]. This interaction is critical to consider when estimating the amount of plastic debris entering the oceans [6]. Hence, there is a knowledge gap in developing models that consider an active bed where the microplastics and sediment particles are transported and deposited following the river hydraulics.

Considering the interactions of sediment–microplastic mixtures in rivers permits the simulation of more realistic scenarios that support the understanding of the consequences of plastic pollution and the location of its sources. Therefore, a numerical hydromorphodynamic river model has been developed to simulate a riverbed where sediment–microplastic mixtures are deposited, eroded, resuspended and transported as a function of river flow. Hydromorphodynamic models are able to simulate the evolution of the bedform shape in time by the erosion, deposition and transport of the suspended or deposited particles. Such a model allows us to explore a set of key outstanding research questions, in particular:

(A) Plastic load balances in the river: How much plastic is entering and leaving the river system?
(B) Spatial and temporal distribution patterns of microplastics: How does the interaction between sediment and microplastics influence the transport and deposition patterns of plastic fluxes.
(C) Effects of plastics on bed morphology: What are the morphological changes in a riverbed with microplastics?

To achieve the objectives of this research, an extant braided river numerical model [28] was selected as the baseline scenario without microplastics. Two suspended microplastic loads were added as initial conditions to create scenarios with plastic. The scenarios were used to analyse the deposited and transported microplastic loads and bedform formation to answer the research questions detailed above. The analysis includes the results of the temporal and spatial distribution of microplastic loads and differences in river morphology, application of the model results, their advantages and disadvantages. Our study suggests a riverbed that acts as a storage site for microplastics near the source, with high areas of deposition in the riverbanks and the inner curve of the channels.

## 2. Methods

Delft3D was used in a depth-averaged (2DH) configuration to recreate an active riverbed where mixtures of microplastic and sediment are interacting to form a braided river. This hydromorphodynamic software package is an established numerical model that uses the Navier–Stokes equation to resolve the unsteady flow [29].

The study case is a calibrated braided river model by Schuurman *et al.* [28]. The model is well-tested and has been used in other research [30–32]. The braided river model was selected since this type of morphology promotes the sinking and fragmentation of plastic in its sediment bars, vegetation, wood jams and migration of the bed [33,34]. The grid size was refined, and the bed sediment particle size was changed from the original model to adjust the initial conditions to the objectives of this research. A summary of the model set-up conditions is listed in table 1.

**Table 1.** Initial and boundary conditions.

parameter	value
inlet flow	20 000 m^3^ s^−1^ (partitioned over 10 cells)
channel width × length	2500 m × 80 000 m
initial bed slope	0.0093%
initial water level	5 m
riverbed sediment size	105 µm
riverbed sediment dry bed density	1650 kg m^−3^
microplastic size	686 µm
microplastic density	1150 kg m^−3^
microplastics suspended load	scenario B = 1000 particles per cubic metre. scenario C = 3000 particles per cubic metre.
grid cells length × width	1202 × 62 rectangles
microplastic transport predictor	Partheniades–Krone formulation [35]
sediment total load predictor	Engelund−Hansen [36]
microplastic fall velocity [37]	15.5 mm s^−1^
microplastic critical erosion [38]	0.055 N m^−2^
microplastic erosion parameter	0.001 kg m^−2^ s^−1^
hydrodynamic simulation time	31 days
morphodynamic simulation time	775 days
study period	42 morphodynamic days, from day 188 to day 229

### (a) Model set-up

**Figure 1.**
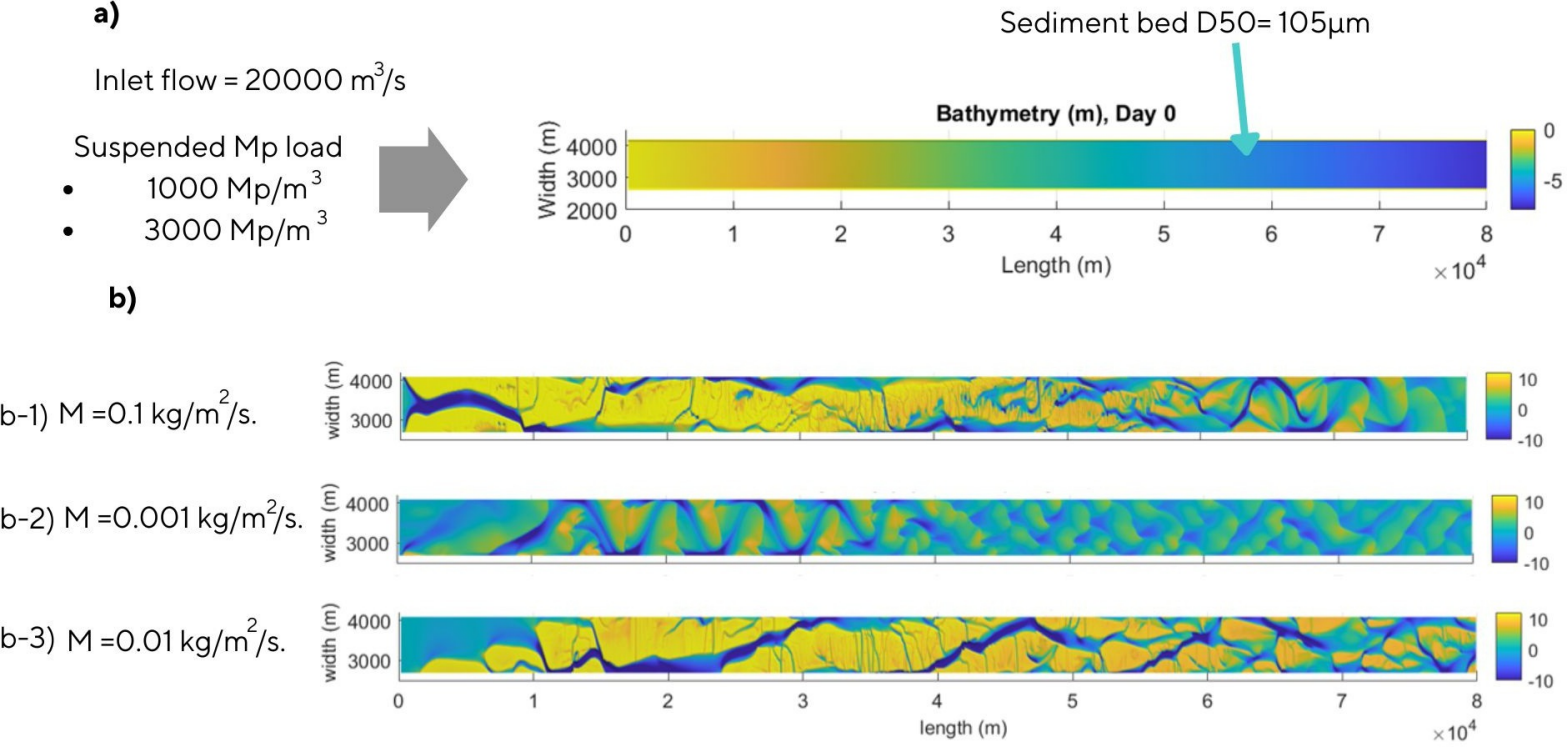
Hydro geomorphological model initial conditions. (a) Initial bathymetry (m), inlet flow and suspended microplastics load. (b) Bed elevation results (m) for the time step day 31 and the erosion parameters (equation (2.1), parameter ‘M’): b−1) 0.1 kg m^−2^ s^−1^, b−2) 0.001 kg m^−2^ s^−1^ and b−3) 0.01 kg m^−2^ s^−1^.

The initial bathymetry of the braided river is a linear channel with a constant slope of 0.0093%, a length of 80 000 m and a width of 2500 m (figure 1*a*). The domain consisted of 62 × 1202 grid cells. The upstream discharge is 20 000 m^3^ s^–1^, partitioned over 10 cells and the initial water level is 5 m. The sediments in the bed had a particle size of 105 µm to represent a finer sand, with a density of 2650 kg m^–3^. The total load predictor of Engelund–Hansen [36] is used to calculate the transport of the sand fraction.

#### (i) Microplastics modelling initial conditions

The microplastics type selected is nylon pellets with cuboid shape, with an average side of 513.17 ± 41 µm and equivalent diameter 686 ± 2.4 µm (measurement obtained with a Malvern Mastersizer machine), and a density of 1150 kg m^−3^. Pellets are primary microplastics used for many industrial applications and are of a type of microplastic shape commonly found as pollutants in the environment [39,40]. The microplastic particles were formulated as a suspended fraction entering the river system at the upstream boundary. The transport–deposition predictor for the load fluxes between the water phase and the bed are calculated with the Partheniades–Krone formulation [35] (equations (2.1)–(2.4)). The formulation can represent the erosion–sedimentation and resuspension of a suspended particle, representing the dynamics of a plastic particle, defined as


(2.1) $$Erosion\;flux,\;E=M\;S\left(\tau_{b},\;\tau_{ce}^{*}\right),\;\;\;\;\;\;\;\;\;\;\;\;\;\;\;\;\;\;\;\left(kg\;m^{-2}\;s^{-1}\right),$$

(2.2) $$S\left(\tau_{b},\;\tau_{ce}^{*}\right)\;\;=\left\{ \begin{array}{l} \left(\frac{\tau_{b}}{\tau_{ce}^{*}}-1\right),\;when\;\tau_{b}>\tau_{ce}^{*},\;\\ 0,\;when\;\tau_{b}\leq \tau_{ce}^{*}, \end{array}\right.\;\;\;\;$$

(2.3) $$Deposition\;flux,De=w{\;}_{s}C{\;}_{b}S(\tau_{b},\tau_{cd}^{*})\left(kg\;m^{-2}\;s^{-1}\right),$$

(2.4) $$S\left(\tau_{b},\;\tau_{cd}^{*}\right)\;\;=\left\{ \begin{array}{l} \left(1-\frac{\tau_{b}}{\tau_{cd}^{*}}\right),\;when\;\tau_{b}<\tau_{cd}^{*},\;\\ 0,\;when\;\tau_{b}\geq \tau_{cd}^{*}, \end{array}\right.\;\;\;\;$$

where *E* is the erosion flux, De is the deposition flux, *M* is the erosion parameter, $\tau_{b}$ bed shear stress, $\tau_{cd}^{*}$ critical deposition shear stress, $\tau_{ce}^{*}$ critical erosion shear stress, $w_{s}$ fall velocity and $C_{b}$ is the average sediment concentration. The particle is considered to be transported in suspension when the bed shear stress ($\tau_{b}$) is higher than critical erosion shear stress ($\tau_{ce}^{*}$) and deposition depends when the bed shear stress ($\tau_{b}$) is lower than the critical deposition shear stress ($\tau_{cd}^{*}$). Lofty *et al*. [41] studied microplastic bedload saltation using microplastics with a mass 70 times larger than that used in this numerical model and still reported that 5% of the microplastics travelled as a suspended load, suggesting that the assumption that the nylon pellet travels as a suspended particle is reasonable.

Researchers have measured the fall velocity and critical shear stress for different microplastic densities, shapes and sizes [42,^43^,^25^,^44^]. The studies can suggest accurate correlations when they include one type of shape and plastic with a range of sizes [42], but when the studies include different variety of shapes, sizes and types of polymers [43,^25^,^44^,^45^]), the errors in the correlations can be higher than 50%. This emphasizes that the parameters of microplastics depend on the dimensionless diameter, shape categories and density of the plastic. For this reason, to estimate the microplastic velocity it was decided to use an empirical formula for particles with non-spherical shapes given by Ferguson & Church [37], and for the particle shear stress the correlation made by Soulsby & Whitehouse [38]. The equations are based on particle (density, shape and size) and fluid physics (density, kinematic viscosity) properties. Hence, using the physical characteristics of the nylon pellets, the particle fall velocity is estimated as 15.5 mm s^−1^ [37], and the particle critical erosion is 0.055 N m^−2^ [38].

Similar to the estimation of the microplastics fall velocity and shear stress, a realistic erosion parameter equation (2.1) was needed to represent the erosion of microplastic from the bed. In the literature, this erosion parameter is not defined for the specific microplastic particle selected. For this reason, the parameter was approximated by creating models with three different values: 0.0001, 0.001 and 0.01 kg m^−2^ s^−1^, and selecting the one that gives a similar morphology to the baseline braided river.

The resulting bathymetry shows three different morphologies (figure 1*b*). The model with an erosion parameter of 0.1 kg m^−2^ s^−1^ creates a sinuous channel, the model with 0.001 kg m^−2^ s^−1^ never converged because the erosion parameter was too high, and all the sediment microplastic were eroded. Finally, the intermediate erosion parameter (0.01 kg m^−2^ s^−1^) was selected as a satisfactory value because it created a braided river morphology similar to the control model. Ballent *et al*. [46] determined this parameter in laboratory conditions for plastic HD pellets with a density of 1055 ± 36 kg m^−3^, obtaining a value of 0.014 kg m^−2^ s^−1^, similar to the value selected and validating the results of the tests.

#### (ii) Model scenarios.

Three main scenarios were selected to achieve the objectives of this research:

(A) The morphodynamic braided river model without plastic. This model is used as a baseline to compare the morphologic changes with the plastic scenarios.
(B) The morphodynamic model with a suspended microplastic load of 1000 particles per cubic metre.
(C) The morphodynamic model with a suspended microplastic load of 3000 particles per cubic metre.

The horizontal spatial distribution of microplastic pollution in rivers is characterized by variable concentrations. Waldschläger *et al.*[ 47] compiled publications on plastic pollution in river systems from 34 rivers and reported maximum concentrations in water samples of 8925 ± 1591 microplastics per square cubic metre (Mp m^−3^) in China and 12 932 Mp m^−3^ in the United States, and a minimum of 1 Mp m^−3^ in the Ottawa River in Canada. For this reason, the suspended concentrations were based on 1000 and 3000 nylon pellets per 1 m^3^ of water flow, counted as 1.94 × 10^–4^ kg m^−3^ (1000 Mp m^−3^) and 5.83 × 10^–4^ kg m^−3^ (3000 Mp m^−3^), similar to a medium concentration of microplastics in rivers [47]. The microplastic load was added as a constant suspended concentration entering the river discharge at the upstream boundary of the model from the beginning to the end of the run time. Based on experimental work [48], the particle interaction in the suspension can be described as being in a free settling regime and non-cohesive. Therefore, the cohesive processes was switched off in the standard model.

All the models were run with a hydrodynamic period of 31 days and a morphological scale factor of 25, resulting in 2.12 morphodynamic years. After running the models, it was decided to select 42 morphodynamic days as the study period, starting on day 188 and ending on day 229. This period was chosen because it was observed to be in approximate morphodynamic equilibrium of a braided river, following a similar approach that was taken by Schuurman [49] and Baar [30].

#### (iii) Model validation

**Figure 2.**
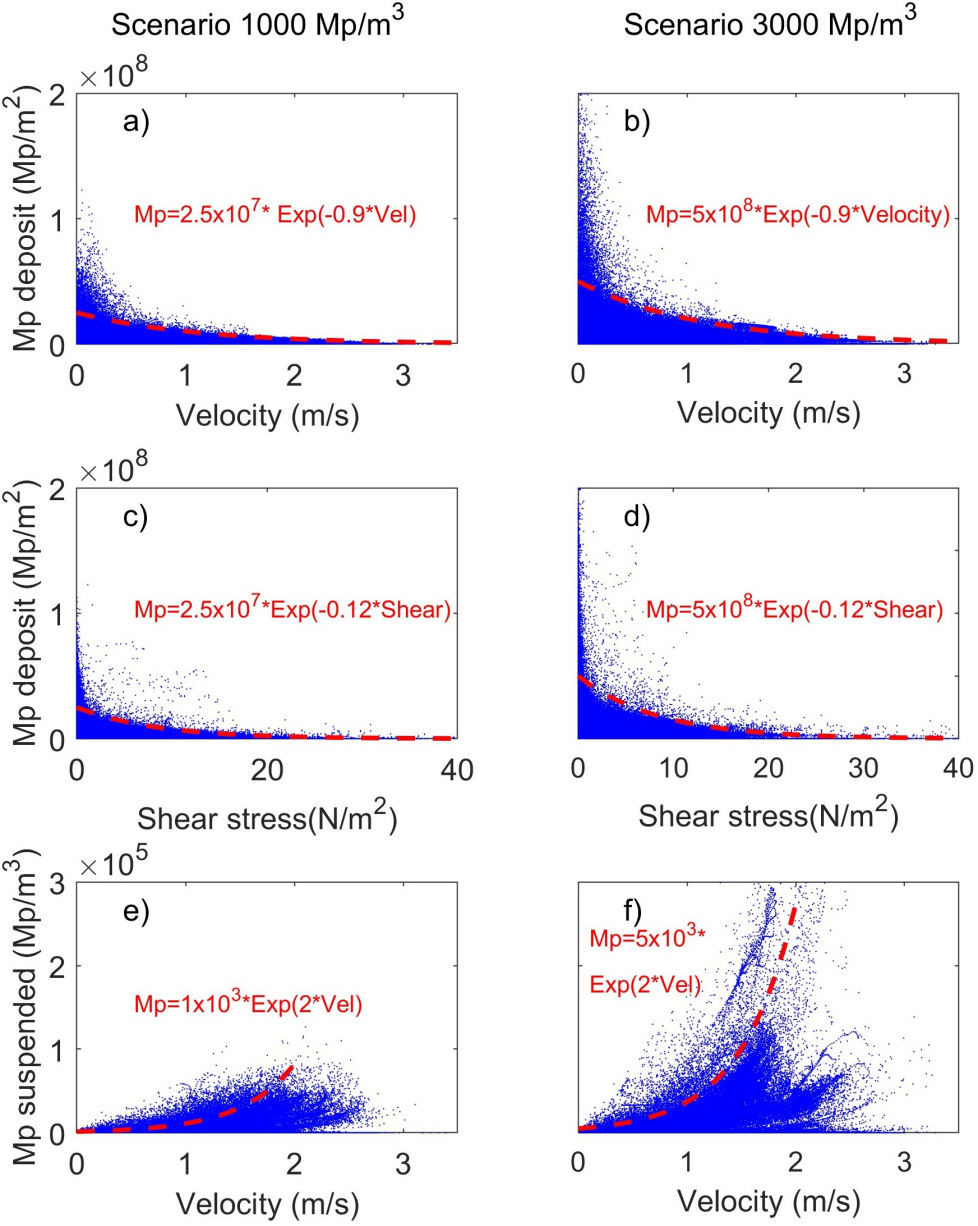
Relations between microplastic loads versus hydraulic parameters. (a,b) Microplastics deposited versus velocity distribution, (c,d) Shear stress versus microplastics deposited. (e,f) Suspended microplastics versus velocity. (a,c,e) correspond to the results for scenario 1000 Mp m^−3^ and (b,d,f) scenario 3000 Mp m^−3^. Red dotted lines correspond to exponential decay curves that best enclose the point cloud.

The results of the velocity and shear stress values versus microplastic loads are shown in figure 2. The exponential decay curve that promotes the best fit to the point cloud shows that both scenarios have the same decay factor, where higher amounts of microplastics are deposited at lower velocity and shear stress, and lower amounts of microplastics are transported at lower velocity. The results are consistent with field measurements and laboratory results, where the fluid dynamics of microplastic particles show a clear relationship to the hydraulic parameters of the flow [10,12–14]. Further discussion of the comparisons of the results with field observations and laboratory experiments is included in the following section.

## 3. Results and discussion

### (a) Temporal distribution of the microplastic load

**Figure 3.**
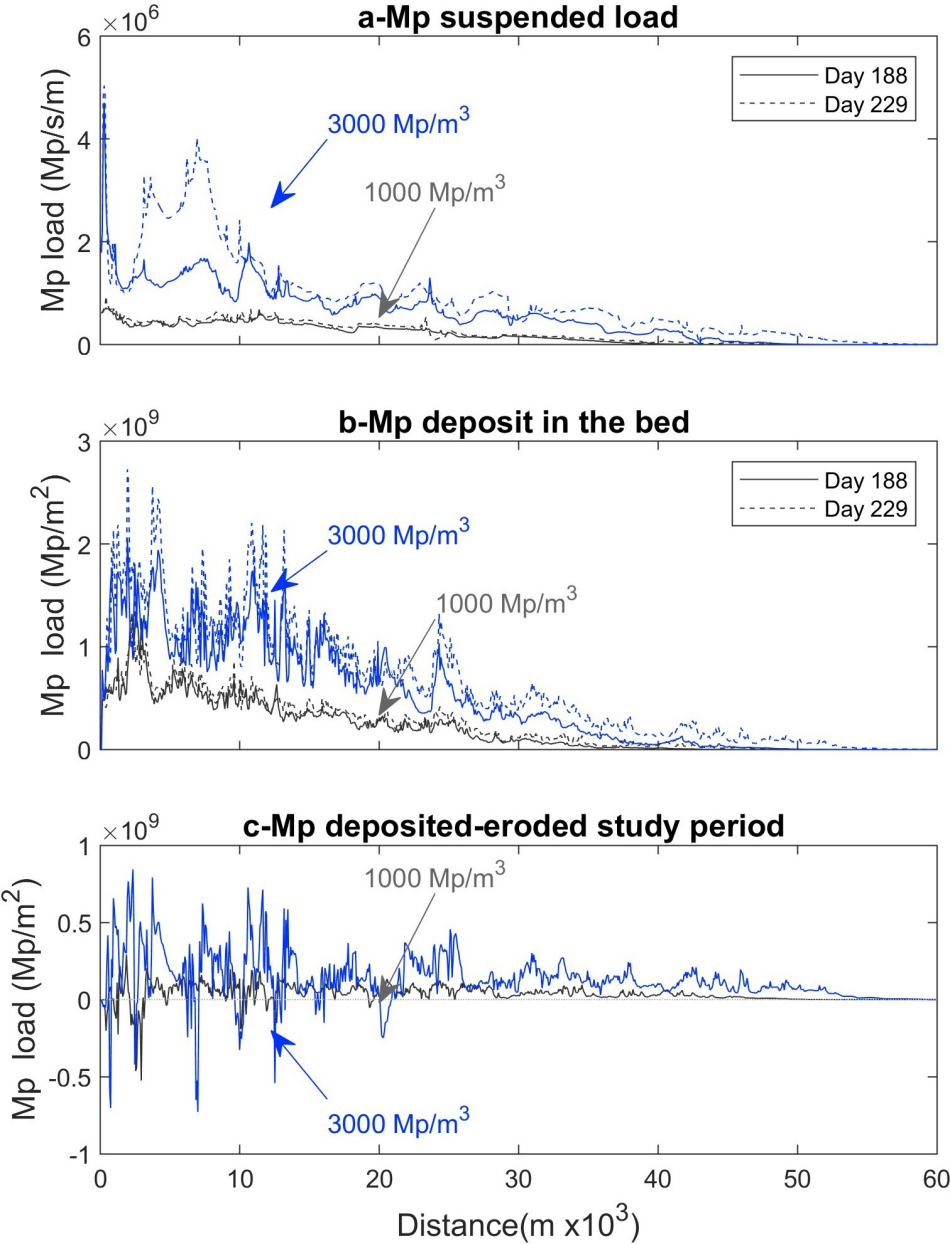
Temporal distribution of microplastics loads: (a) suspended (b) deposited from day 0 to day 188−229 and (c) deposited-eroded between day 188 and 229; scenario with 1000 Mp m^−3^ (black) and 3000 Mp m^−3^ (blue).

Plotting the suspended and deposited microplastic load versus the length of the river represents the load entering the model and the rate at which it is stored in the sediment bed, eroded and resuspended (figure 3). The results showed that the microplastic moves downstream over time, an effect of the suspension, deposition and resuspension of plastic in the system. The suspended microplastic load shows different peak loads in the first 10 km, showing a more robust load in resuspension, more noticeable in the scenario with 3000 Mp m^−3^ (figure 3*a*). The microplastic load deposition in the sediment bed per m^2^ (figure 3*b*) shows a decreasing tendency as an effect of the suspended microplastic load deposited and stored in the sediment bed. The microplastic deposition curves have a similar trend at the beginning (day 188) and end of study period (day 229), describing a higher deposition rate in the sediment bed at the beginning of the model and a more constant deposition rate after 30 km. The microplastic load eroded (negative values) over 42 days represents the resuspension of microplastic deposits from previous time steps (figure 3*c*).

The model showed that higher amounts of microplastics in the river accelerate the transport rate of the suspended and deposited load. The microplastics move at an average rate of 140 m day^−1^ for the 1000 m^3^ s^−1^ model and 173 m day^−1^ for the 3000 m^3^ s^−1^ model, travelling between 6 and 7 km in 42 days under average flow conditions.

The total suspended microplastic load over 42 days sets the microplastic flux balances for the study period. For the 1000 Mp m^−3^ scenario, it is estimated that 84.08 billion particles entered the braided river, of which 100% of the load was deposited on the riverbed. A total of 2980 billion particles were in suspension, with an estimated resuspension of 35 times the total load. A total of 310.17 billion particles entered the braided river with 3000 Mp m^−3^. Of this, 100% of the load was deposited in the riverbed and 35,835 billion particles were in suspension, estimating a resuspension of 116 times more than the total load. This result suggests that the numerical model of plastic pollution defines a riverbed where the microplastic is deposited, eroded and resuspended, with high storage near the inlet boundary (release point) and slow transport downstream.

The transport of particles in suspension, high deposition of plastic near sources and slow transport downstream have been found in studies of microplastic pollution. Koutnik *et al.* [50] analysed the microplastic concentration reported in 196 studies from 49 countries. They described that microplastic concentration are higher in rivers than at the coast, reflecting the storage capacity of microplastic loads in the river system. Corcoran *et al*. [51], based on the study of sediment layers in a lake, found that microplastics have been deposited in the deepest region for less than 38 years. Nizzetto *et al.* [52] created a mathematical model of the Thames River, noting that non-buoyant microplastics larger than 0.2 mm are contained in the sediment and resuspended during high flows. The Brisbane River (Australia) was modelled by He *et al*. [53] with a three-dimensional particle transport model. In the model, high-density plastics accumulate near to the source points, high velocity promotes the transport of the sinking microplastics in the bed, and describes a slow dispersion and transport of the microplastics in the river. Ballent *et al*. [46], in their numerical models of plastic pollution, also mentioned significant accumulation of plastics near the release points.

The results have implications for estimating how much plastic enters the oceans from the river system per year, confirming that the system-wide mobilization of plastics in rivers is limited [50,53,54]. The use of mathematical models has been essential to attempt to predict how much plastic is transferred from the river to the ocean using simplifications of the transport and deposition of the plastic in the rivers [5,6]. Model validation suggests that unpredictable data could be related to rivers acting as sinks for plastic [6]. This emphasizes the importance of considering sediment–microplastic interactions in the riverbed, to reflect the high storage capacity of the plastic near its sources and its slow transport.

### (b) Spatial distribution of the microplastic load

**Figure 4.**
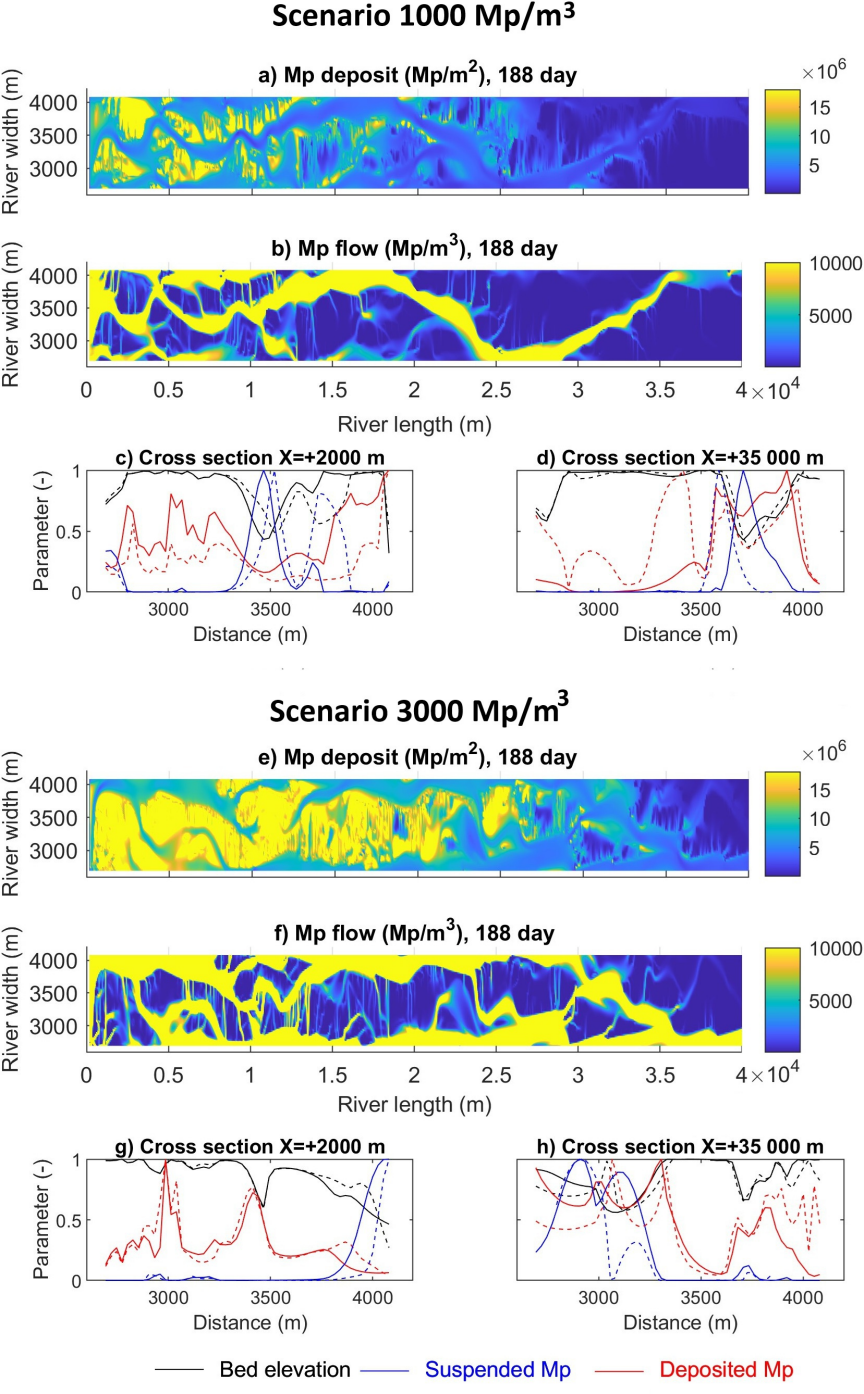
Spatial distribution of microplastic loads in the braided river. Deposited microplastic loads in the riverbed for scenarios 1000 Mp m^−3^ (a) and 3000 Mp m^−3^ (e) . Suspended microplastic loads in the river flow for scenarios 1000 Mp m^−3^ (b) and 3000 Mp m^−3^(f) . Bed elevation, suspended and deposited microplastic load cross sections, for scenarios 1000 Mp m^−3^ (c,d) and 3000 Mp m^−3^ (g,h). The solid line corresponds to day 188, and the dashed line to day 222.

The spatial distribution of microplastic deposition and transport for the braided river is shown in figure 4*a*,*b*,*e*,*f*, highlighting in yellow the highest amount of plastic deposited at the beginning of the river and the highest suspended microplastic fluxes travelling in the main channels. Based on the model bed elevation and the distribution of deposited microplastics, two specific patterns can be seen at the top of bars and in channels. The top of the bars can be considered as areas with unitary elevations from 1 to 0.9 and the channels with elevations lower than 0.9 m.

The microplastics are deposited following the pattern of the bathymetry, with some peaks in the channels (figure 4). It is estimated that 45%–52% of the microplastics were deposited at the top of the bars, 45% in the lateral channel slopes (0.6−0.9 unitary elevations) and the remaining 3%–10% in the deeper channel elevations (below 0.6 unitary elevations). The highest amounts can be found at the beginning of the river, and as time passes the microplastic reduces its presence in the highest elevations (figure 4*a*,*b*,*e*,*g*). Significant changes can be clearly seen when comparing the 2 km with 35 km cross sections, where the deepest elevations show the least microplastic load deposited in the bed (figure 4*c*,*d*,*g*,*h*). In some cases, a peak load (microplastic hot spot) is formed in one of the lateral banks of the channels (e.g. at distances 3000 and 3400 m in the cross section in figure 4*g*.)

Ninety-five per cent of the suspended microplastic load was transported in the channels and secondary channels. Downstream, the microplastic load accumulated more in the deep channel (main channel), and less microplastic was transported in the secondary channels, and the load is also transported in the crossbar channels over time (figure 4*b*–*f*)

The spatial distribution highlights that the microplastics can be deposited everywhere, but hotspots are present as extended areas, with the effect of retention in depositional layers. The results can be related to the analysis of field samples, in which researchers describe microplastic deposits in the riverbanks with a highly heterogeneous lack of consistency [16,55]. These results suggest that the best way to find a deposition pattern is to use cross-sectional sampling, which has already been recommended by Haberstroh *et al*. [56].

**Figure 5.**
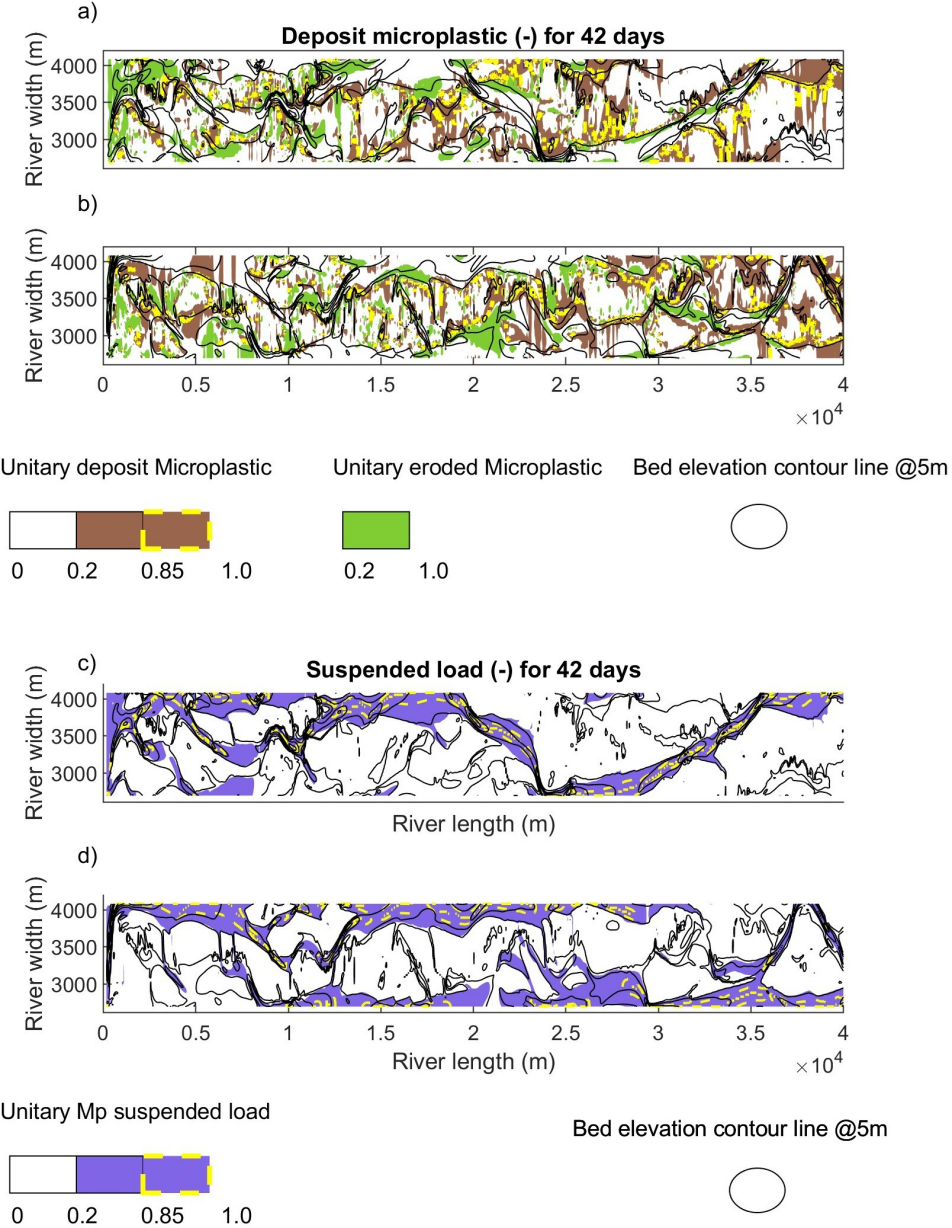
Total microplastics deposited, eroded and suspended for 42 days (study period). Deposited and eroded microplastics for the 1000 Mp m^−3^ (a) and 3000 Mp m^−3^ (b) scenarios. The green areas represent the eroded microplastics, the white areas the lower microplastic deposits, the brown areas the intermediate microplastic deposits, and the brown-yellow dashed areas the higher microplastic deposits. Microplastic suspended load for the 1000 Mp m^−3^ (c) and 3000 Mp m^−3^ scenarios. The white areas transport the lower suspended load, the blue areas the medium suspended load and the blue-yellow dashed areas the highest suspended load. The dark grey lines are the riverbed elevations every 5 m.

To investigate the microplastics deposited during the study period, it was decided to extract the deposited microplastic load from day 188 to day 229, as the daily analysis (figure 3*a*) showed that a percentage of the suspension corresponded to previous time steps. The analysis (figure 5*a*–*c*) shows that the microplastics were deposited on the bars and riverbanks, with the highest amounts on the inner curve of the main channel, areas recognized as depositional zones with lower velocities [57,58]. The same patterns have been found in field samples by Rezende-Gerolin [59], who described that the lowest concentrations of microplastics in the Amazon River are near the erosive areas, especially in the thalweg. Mani *et al*. [60] described higher retention of microplastic particles in the right bank of the Rhine river delta, in the area of lowest flow velocity, where sedimentation rates increased. Similar effects have been measured by the effect of vegetation and cohesive sediments [61]. The lowest amount was deposited in the deeper areas of the channels, areas with higher velocities.

The higher suspended load travels in the main channel of the braided river (figure 5*c*,*d*) and then enters the crossbar channels; consequently, microplastic loads are higher in the main channel than in the secondary channel. In terms of spatial distribution, the high suspended microplastic load is found in the thalweg of the main channel, which has been recognized as the area with higher velocities and more extensive transport of microplastics [53]. Finally, the model explains that the effects on the microplastic spatial distribution are related to the hydrodynamic conditions, sediment particle size and flows, consistent with other studies [62,63].

**Figure 6.**
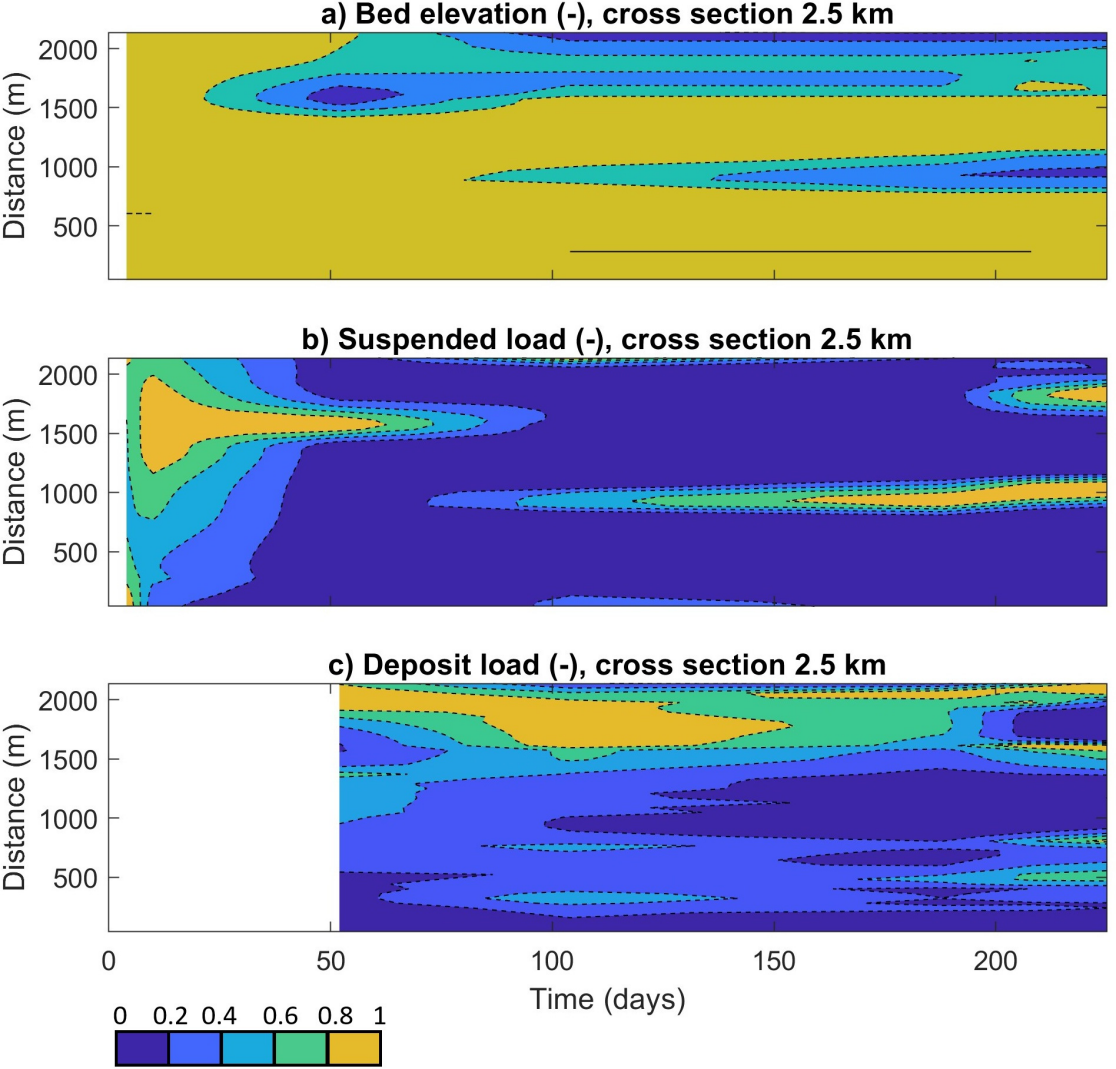
Normalized depths (a), suspended microplastic load (b) and deposited microplastic load (c) over time for the 2.5 km river cross section, 1000 Mp m^−3^ scenario.

The highest amount of microplastics transported in the channels explains that most of the particle deposition on the bars is a consequence of previous time steps before day 188 (Figure 4b-f). To investigate this effect, the temporal evolution of the unitary bed elevation, suspended and deposited microplastic load were plotted for the 2.5 km cross section (1000 Mp m^−3^ scenario) for days 4, 10, 52, 104, 188, 208 and 229 (figure 6).

The evolution of these cross sections showed a suspended load moving across the width of the model from day 0 to 10 (figure 6*b*). On day 10, the method started to form a channel in the 1500 m distance axis until day 313 (figure 6*a*). A second channel is formed from day 52 to day 229 in the 1000 m distance axis (figure 6*a*). Comparing the suspended load with the bed elevation, it is clear how the microplastic fluxes started to concentrate in the second of the two channels from day 52 to day 229 (figure 6*b*), as the first channel is filled. The deposited microplastic load is distributed over the whole 2.5 km cross section from day 52 to day 229 and can be concentrated in the main channels or on the bars (figure 6*c*). The evolution of the loads versus the bed elevation explains how the model reproduced the depositional processes of the mixed layer of plastic and sediment during whole run.

### (c) River morphology differences

**Figure 7.**
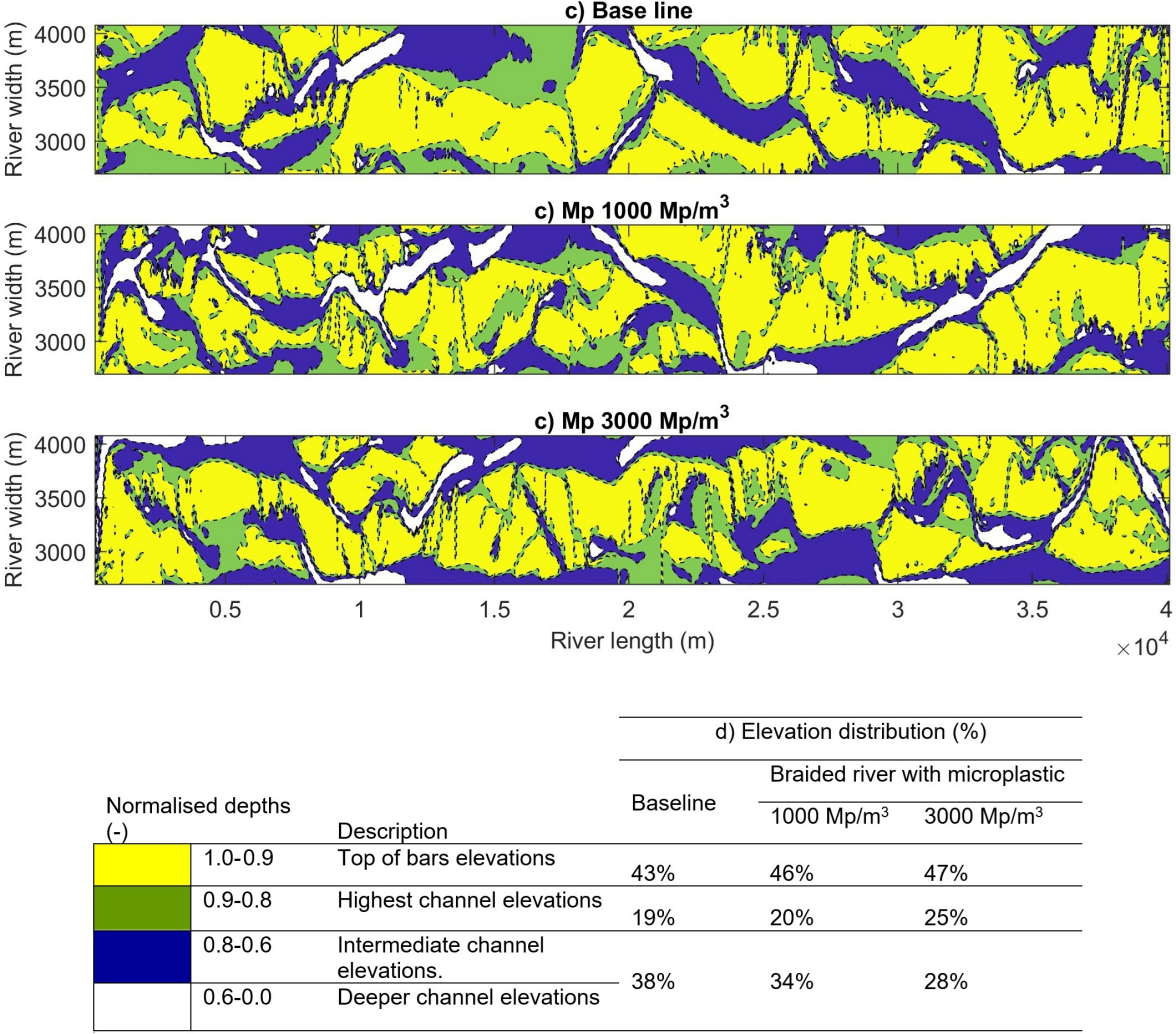
Bathymetry of the braided river without (a) and with (b,c) plastic scenarios. The yellow represents the top of the bars, the green the banks or cross-bar channels, the blue the mid-channel elevations and the white the deeper channel elevations. (d) Bed elevation distribution in the first 40 km of the river.

Modelling the scenarios with and without plastic resulted in three different river morphologies, showing that the erosion and deposition capacity of the river flow is affected by the initial plastic load and produces changes in bar morphologies (figure 7). The results are due to the fact that the software methods take into account erosion rates which are proportional to the bed composition [64].

The high amount of microplastics deposited at the beginning of the model results in deeper channels and larger bars. In the 1000 particles/m^3^ scenario, the plastic modified the erosion rates in the sediment bars and riverbanks, resulting in deeper channels. In the 3000 particles/m^3^ scenario, the amount of plastic deposited in the bed was so high that the model was unable to generate a braided river planform. Instead, a single channel was formed to the left of the grid. Downstream, the model simulates three different morphologies, a product of the initial conditions and the presence or not of the microplastics. Comparing the results with and without plastic shows how the effects of the microplastic load changed the bed elevation distribution (figure 7*d*).

The estimated changes in the morphology of the braided river are calculated indirectly using the erosion parameter and the shear stress in the Partheniades–Krone formulation [35]. The equations allow the model to reproduce the effects of the plastic in the sediment bed, where a high erosion parameter promotes higher erosion rates and lower values promote less erosion. The model replicates results for morphological changes, where the plastic increases the mean diameter of the material in the riverbed and a higher critical shear stress is required to erode the bed [25,26], extending the area of the bars and promoting deeper channels.

Russell *et al*. [19] used flume experiments to study the interaction of microplastics and sediment in the riverbed. In the study, heterogeneous distributions of plastic deposits were formed in the sediment, resulting in an increase in plastic distribution in the outer side of the dune (stoss side) and a slow formation of the inner deposition side (lee side) [19]. The same effect was observed on a larger scale in the numerical models, with high plastic deposition filling the shallower channels (figure 7), forming plastic deposits and affecting the lateral evolution of the channel morphology.

### (d) Model advantages and limitations

The main advantage of the multiphase sediment–microplastic transport model is its ability to simulate a riverbed where a microplastic load and sediment particles exchange materials from the bottom computational layer to the bed and vice versa. The model simulated the changes in erosion rates as a result of the microplastic in the sediment bed. The model assumed that the erosion rate is proportional to the availability of the sediment fraction considered in the top-most layer of the bed stratigraphy, suggesting that the model may be useful for identifying the sources of industrial plastic emissions, which is an important indicator of plastic pollution (65). Our model treats a plastic particle as another sediment particle, in agreement with the newly proposed framework to unify the growing ambiguity in microplastic description and identification [66] and as such, relies on physical principles long-established in the field of sedimentology that help us look at plastics in a very solid framework.

The limitations of the multiphase sediment transport model are related with the erosion parameters, bed load transport predictor and microplastic aggregation processes. Further work is recommended to estimate properly the erosion parameters for the specific microplastic and sediment mixtures under controlled laboratory conditions; key parameters that shape the morphology of the river. However, the variety of microplastic densities, shapes and sediment sizes generates a range of erosion parameter scenarios limiting the accessibility of the method. The Partheniades–Krone formulation [35] is based on the deposition and resuspension process and does not consider if the particle is transported as suspended, rolling–sliding, or saltating. Particles transported as rolling–sliding or by saltation are described in sediment transport as bed load, and have been observed in plastic particles in flume experiments with microplastics [ 41] and microplastics–sediment mixtures [19]. This highlights the need to define the physical properties (shape, density, size) and hydraulic conditions (shear stress, velocities, turbulence) under which plastic particles are transported as suspended, bedload concentration or any other new processes that may need to be defined for the variety of microplastic shapes. Finally, aggregation cannot be integrated when using this method [22,67,68].

## 4. Conclusion

The reported research recreated a numerical model with the dynamics of deposition, erosion, resuspension, and transport of negatively buoyant microplastics in a braided river, where the sediments and microplastic particles interact as a function of the river flow. The artificial braided river simulated a sediment bed that acts as a source of storage for microplastics near the point of release, limiting their availability to be resuspended and transported downstream. The high deposition of microplastics increases the capacity of the river flow to erode the sediment bed, resulting in deeper channels and larger river bars. The highest amounts of microplastics were deposited in the inner curve of the main channel in its banks, and the highly suspended microplastic load is transported in the thalweg of the main channel. Simulations show that microplastics can be deposited and stored everywhere within a river, with localized hotspots of high microplastic concentration related to flow dynamics.

The model demonstrates the importance of the interaction between sediments and microplastics in the riverbed, as a more accurate estimation of the total load of plastic that a river transports. The model highlights the importance of considering the river flow dynamics in studies of the spatial distribution of microplastic loads, and the capacity of plastic loads to modify the riverbed morphology. Key areas where models can be improved include: development of positively buoyant microplastic particle transport models; integration of sedimentary depositional layers; development of microplastic erosion models; dynamics of microplastic and mixed natural (non-cohesive and cohesive) sediment suspensions; and validation of the results with detailed laboratory or field data. Further studies are needed to determine plastic–sediment erosion parameters and the threshold(s) at which microplastics are travelling in suspension or bedload.

## Data Availability

This article has no additional data.
